# Introduction of Androgen Receptor Targeting shRNA Inhibits Tumor Growth in Patient-Derived Prostate Cancer Xenografts

**DOI:** 10.3390/curroncol30110683

**Published:** 2023-10-24

**Authors:** Patrick B. Thomas, Saeid Alinezhad, Andre Joshi, Katrina Sweeney, Brian W. C. Tse, Gregor Tevz, Stephen McPherson, Colleen C. Nelson, Elizabeth D. Williams, Ian Vela

**Affiliations:** 1 School of Biomedical Sciences at Translational Research Institute (TRI), Faculty of Health, Queensland University of Technology (QUT), Brisbane, QLD 4102, Australia; pb.thomas@qut.edu.au (P.B.T.);; 2 Australian Prostate Cancer Research Centre—Queensland, Brisbane, QLD 4102, Australia; 3 Queensland Bladder Cancer Initiative (QBCI), Brisbane, QLD 4102, Australia; 4 Department of Urology, Princess Alexandra Hospital, Brisbane, QLD 4102, Australia; 5 Preclinical Imaging Facility, Translational Research Institute (TRI), Brisbane, QLD 4102, Australia; brian.tse@tri.edu.au; 6 Centre for Genomics and Personalised Health, Queensland University of Technology (QUT), Brisbane, QLD 4000, Australia

**Keywords:** prostate cancer, organoids, precision medicine, patient-derived xenograft, androgen receptor

## Abstract

Patient-derived xenograft (PDX) models have been established as important preclinical cancer models, overcoming some of the limitations associated with the use of cancer cell lines. The utility of prostate cancer PDX models has been limited by an inability to genetically manipulate them in vivo and difficulties sustaining PDX-derived cancer cells in culture. Viable, short-term propagation of PDX models would allow in vitro transfection with traceable reporters or manipulation of gene expression relevant to different studies within the prostate cancer field. Here, we report an organoid culture system that supports the growth of prostate cancer PDX cells in vitro and permits genetic manipulation, substantially increasing the scope to use PDXs to study the pathobiology of prostate cancer and define potential therapeutic targets. We have established a short-term PDX-derived in vitro cell culture system which enables genetic manipulation of prostate cancer PDXs LuCaP35 and BM18. Genetically manipulated cells could be re-established as viable xenografts when re-implanted subcutaneously in immunocompromised mice and were able to be serially passaged. Tumor growth of the androgen-dependent LuCaP35 PDX was significantly inhibited following depletion of the androgen receptor (AR) in vivo. Taken together, this system provides a method to generate novel preclinical models to assess the impact of controlled genetic perturbations and allows for targeting specific genes of interest in the complex biological setting of solid tumors.

## 1. Introduction

Prostate cancer (PCa) is the most common lethal cancer in men and represents a major cause of cancer-related morbidity and mortality worldwide [1,2]. It is a relatively slowly growing cancer and, when localized, is highly curable with a five-year survival rate close to 100% [3,4]. Unfortunately, once PCa metastasizes, it becomes incurable. Androgen deprivation therapy (ADT) is used as a first-line therapy to treat patients with metastatic PCa; however, it is not curative, and the disease ultimately progresses to castration-resistant prostate cancer (CRPC) with a median survival of approximately 40 months [5]. While survival rates have improved over recent years as a result of the second-generation anti-androgen therapies enzalutamide and abiraterone, the tumors inevitably become resistant to these therapies, and thus, a significant number of prostate cancers remain incurable and lethal [6]. Advances in genomic and transcriptomic profiling of clinical samples have revolutionized our knowledge of the molecular drivers of PCa [7,8,9]. The development of effective treatments for advanced PCa has been hindered, however, by a lack of reliable and relevant preclinical models. Consequently, there is a high failure rate for novel therapies during translation from the laboratory to the clinic. Traditionally, cancer cell lines established decades ago have been widely used toward drug discovery and other high-throughput screening efforts due to their relative low cost, ease of use, and biological reproducibility. Although cell lines are an important resource for identifying predictors of treatment response and resistance [10,11], cancer cell lines currently available for in vitro testing are associated with significant limitations. These cell lines have been isolated and cultured from specific tumor subsets under selective in vitro culture conditions. The selection process associated with the establishment of cell lines in monolayer culture results in models that are typically poorly representative of the diversity of human tumors [12] and lack the heterogeneity observed in PCa patients’ tumors. These models also lack the complex biological interplay that occurs in the in vivo setting [13,14]. PCa has also been extremely challenging to grow ex vivo when compared to other cancer types, with few established immortalized cell models widely available for use—severely limiting the range of clinical relevance. Consequently, there is a need for a model that mimics human PCa pathophysiology more closely and reflects the broad genetic and phenotypic spectrum of the disease to advance drug discovery and development.

Patient-derived xenografts (PDXs) have been established as important preclinical cancer models to overcome limitations associated with the use of cancer cell lines. PDXs are grown in immunocompromised mice and retain the genetic and epigenetic aberrations present in the original patient tumors [14,15,16]. This allows investigators to obtain preclinical results that more accurately reflect the clinical response in patients when compared to well-established monolayer cell lines. Similar to the experience with generating prostate cancer cell lines [17], prostate tumor PDXs have proven relatively difficult to establish [14,18]. Several groups have successfully developed PCa PDXs [18], including bone metastasis-derived BM18 and the LuCaP models [19,20,21,22], which represent a wide spectrum of genomic heterogeneity, as well as a variety of pathologic subtypes and treatment histories. A common limitation with these PDX models, however, has been the difficulty in genetically modifying or targeting specific genes of interest in order to further investigate potential therapeutic targets and mechanisms for treatment resistance. PCa PDX models are also limited by several other factors, including a lack of sustained growth in vitro, a lengthy latency period, variable initial engraftment rates of clinical material, and high costs of animal maintenance [14]. While PDX cells can be grown in vitro as 2D and 3D cultures for short periods of time, most PDX-derived cell lines stop proliferating and undergo senescence within a short time [23]. These drawbacks have limited the application of PDXs for drug discovery and high-throughput screening studies. Various in vitro approaches have been described to culture cell lines, primary adult cells, stem cells, and progenitor cells in 3D to develop multicellular entities, which can self-renew, self-organize, and differentiate. The in vitro organoid culture method described by Gao, Vela et al. successfully generated long-term cultures of PCa organoids from metastatic biopsy specimens and circulating tumor cells [24]. Organoids established using this technology retain the genomic and phenotypic features of the patient tumor and are well-suited to in vitro manipulation. This approach effectively addresses several limitations of PDXs as preclinical models. Organoids offer the advantage of rapid, reproducible establishment and scalability, enabling quicker research and drug development timelines. Furthermore, compared to PDX, organoid technologies are also more cost-effective and accessible to a wider range of research institutions [25]. Additionally, this reduces the dependence on patient samples or PDX establishment, which can be limited in a clinical setting [25].

To address and overcome the limitations described above, we used the approach described by Gao, Vela et al. [24] to convert the BM18 and LuCaP PDX models into in vitro organoid cultures in order to generate stable models expressing a reporter gene. As a proof of principle of experimental utility, we manipulated the expression of the androgen receptor (AR), a gene central to prostate cancer biology. This study represents a novel application of a previously reported organoid technology that overcomes some of the limitations of PDXs as preclinical models, increasing their utility and facilitating their application in drug discovery and high-throughput screening.

## 2. Materials and Methods

### 2.1. Generation and Cultivation of Organoids from LuCaP35 and BM18 Xenografts

Experiments using SCID and NSG mice were approved by the University of Queensland Animal Ethics Committee (TRI/QUT/370/17, QUT/TRI/305/15/MRTA/APCRC) and conducted in accordance with the Australian Code for the Care and Use of Animals for Scientific Purposes. Mice were housed under specific pathogen-free conditions in individually ventilated cages (Techniplast, West Chester, PA, USA) at room temperature (20–23 °C), 40–60% relative humidity, and under a 12 h light/dark cycle. Fresh LuCaP35 and BM18 PDX tumors were obtained at the time of routine passage in SCID mice (Animal Resources Centre, Murdoch, WA, Australia). After removal from mice, tumors were immersed in sterile, serum-free prostate culture media (PCM) [24], which contains multiple constituents, including growth factors, noggin, and R-spondin (Supplementary Table S1). PDX tissue was finely minced into 20 to 30 mm^3^ pieces using a scalpel blade and enzymatically digested using collagenase type I (5 mg/mL; Invitrogen, Waltham, MA, USA) in PCM at 37 °C for one hour with gentle shaking. Digested tissue was passed through a 70 µm cell strainer to eliminate macroscopic tissue pieces. The flow-through was centrifuged at 211× *g* and resuspended in fresh PCM media. Dissociated cells and cell fragments were cultured in 12-well low attachment plates. Media was changed twice per week.

### 2.2. Lentiviral Transduction of Xenograft-Derived Organoids

As proof of principle, dissociated LuCaP35 and BM18 cells were transduced using a pGIPZ lentiviral vector (Dharmacon, Lafayette, CO, USA) with a hairpin targeting firefly Luciferase and expressing Turbo Green Fluorescent Protein (GFP) (ThermoScientific, Waltham, MA, USA) following the manufacturer’s protocol. In vitro GFP-labeled cultures were initially validated for GFP expression using fluorescent microscopy imaging on the Nikon Eclipse Ti2-U (with a 470/40 excitation filter and a 535/50 emission filter for GFP visualization). Successfully transduced cells (1 × 10^5^ cells in 100 μL Matrigel™ (Corning Inc., Corning, NY, USA)) were then injected subcutaneously on the flank of six-week-old male NSG mice (Animal Resources Centre). Tumor formation was monitored twice weekly, and tumor volume was measured using digital calipers. Tumor volume was calculated using the ellipsoid formula (L × W^2^ π/6) [26]. Tumor doubling time was calculated using the formula (time × LN(2))/LN(final volume/start volume) [27]. Expression of GFP was monitored using live in vivo bioluminescent imaging (IVIS spectrum, PerkinElmer, Waltham, MA, USA). 

### 2.3. Lentiviral Transduction of LuCaP Organoids to Knockdown AR

For targeted genetic manipulation of xenograft-derived cells, AR knockdown was performed using cells derived from LuCaP35 PDX and a pTRIPZ (Dharmacon) plasmid targeting AR (shAR) [28]. Doxycycline-inducible Turbo Red Fluorescent Protein (RFP) and shRNA were part of a single transcript arising from this vector, which allowed the fluorescent labeling of shRNA-expressing cells. A puromycin drug resistance marker was used for selecting stably transfected cell lines. The lentivirus particles were produced in HEK-293T cells transfected with pCMV-Δ8.2R, pCMV-VSVG (Cell Biolabs, San Diego, CA, USA), and pTRIPZ-shRNA-containing plasmids. Transduction was performed using the protocol described by Van Lidth de Jeude and colleagues for intestinal organoids [29] with the following modifications. Forty-eight hours after seeding xenograft-derived cells, stable transduction with lentiviral soup in the presence of protamine sulphate (8 µg/mL) was performed to generate the LuCaP35-i-shAR organoid line. Cells plated in wells of a 24-well plate were incubated for 24 h in the presence of the virus, after which the media was replaced with fresh PCM media. To permit the cells to recover from viral infection, the organoids were cultured in PCM media for one week before commencing the selection process. The stably transduced cells were selected by treating the cells with puromycin (1 µg/mL) for one week. To confirm lentiviral transduction success and visualize transduced cells, doxycycline (750 ng/mL) was added to organoids following antibiotic selection. Five days after adding doxycycline, fluorescent images of organoids were collected using a Nikon Eclipse Ti2-U inverted microscope. For visualization of GFP, a 470/40 excitation filter and a 535/50 emission filter were used, while for RFP, a 560/40 excitation filter and a 630/60 emission filter were used.

### 2.4. Establishment of LuCaP35-i-shAR Organoid Derived Xenografts

To evaluate the tumor-forming capability of LuCaP35-i-shAR cells, organoids were collected, and viability was measured using propidium iodide (PI), Hoechst 33342 staining [30], and a Nikon Eclipse Ti2-U inverted microscope. Hoechst was used to label the total number of cells as it can readily cross cell membranes and stain DNA of living and dead cells. By contrast, PI only enters cells with compromised plasma membranes, thus selectively labeling dead cells. Organoids were resuspended in 100 µL of cold Matrigel^TM^ and injected subcutaneously on the flank of six-week-old male NSG mouse, as described above. Tumor formation was monitored and measured as described previously.

### 2.5. Passaging LuCaP35-i-shAR Xenografts

Once LuCaP35-i-shAR tumors reached approximately 1 cm^3^, routine passage was performed as per protocol. A proportion of the tumor was fixed in 10% neutral buffered formalin (Australian Biostain, Traralgon, VIC, Australia) and processed for histological and immunohistochemical analyses. An additional piece was stored in PCM media for organoid culture, and the remainder was divided into smaller pieces and re-implanted subcutaneously into new recipient mice. LuCaP35-i-shAR PDX was passaged three times sequentially to evaluate the stability of lentiviral transduction and genetic manipulation.

### 2.6. In Vivo Induction of AR Knockdown

Once the tumor volume exceeded 300 mm^3^, the mice were randomized into two groups. One group had doxycycline added to their drinking water (2 mg/mL) to induce shAR expression, and the other group continued with doxycycline-free water as a control group. The impact of shAR expression on tumor growth was evaluated by measuring the tumor volume twice weekly and collecting weekly fluorescent imaging. An IVIS spectrum CT (PerkinElmer, Shelton, CT, USA) was used to visualize RFP production and evaluate the impact of shAR expression on tumor growth. Briefly, mice were anesthetized (inhalation of ~4% *v*/*v* vaporized isoflurane) and placed inside the imaging chamber with the tumor flank facing the camera. For fluorescent detection, epi-illumination was used to image the tumors. The dsRED filter was used for imaging. Mice were treated with doxycycline water (2 g of doxycycline and 50 g of sugar in one liter of water, with the solution replaced two times per week) or control water. After three to four weeks of treatment with doxycycline/control water, mice were anesthetized using isoflurane, and a terminal bleed was performed prior to tumor collection. Tumors were weighed, and a part of each tumor was fixed in 10% neutral buffered formalin (Australian Biostain, Australia) for histopathological analyses. Another portion of the tumor from each mouse was flash-frozen in liquid nitrogen. Small pieces of tumor (~5 mm^3^) from one untreated (vehicle) mouse were passaged into new recipient mice. 

### 2.7. In Vitro Culture of LuCaP35-i-shAR Xenograft and Induction of AR Knockdown

To assess the organoid formation capability of LuCaP35-i-shAR xenografts and to determine the effect of doxycycline treatment on the level of AR in vitro, xenograft tissue was digested in collagenase as described above, and the resultant dissociated cells were cultured in low attachment 24-well plates. After 72 h, once organoids were formed, doxycycline (750 ng/mL) was added to PCM media in half of the wells to induce AR knockdown, with the remainder of the wells left as untreated controls. Five days after adding doxycycline, fluorescent images of the organoids were collected as described above.

### 2.8. Histological Analysis of Xenograft Tissue and Organoids

For histological analysis, tissues were fixed in 10% neutral buffered formalin overnight before being changed into 70% *v*/*v* ethanol and stored at 4 °C until processed for histology. Sections were dehydrated through a graded series of ethanol and xylene, before being embedded in paraffin as per standard protocols at the Translation Research Institute Histology Facility. Following embedding, 5 µm sections were prepared using a Leica Microtome RM2235 (Leica Biosystems, Nußloch, Germany), mounted onto glass Menzel Superfrost slides (Thermo Fisher Scientific), and subjected to routine hematoxylin and eosin (H&E, Baton Rouge, LA, USA) staining procedures.

## 3. Results

### 3.1. In Vitro Culture of LuCaP35 Xenografts and Lentiviral Transfection

**Figure 1:**
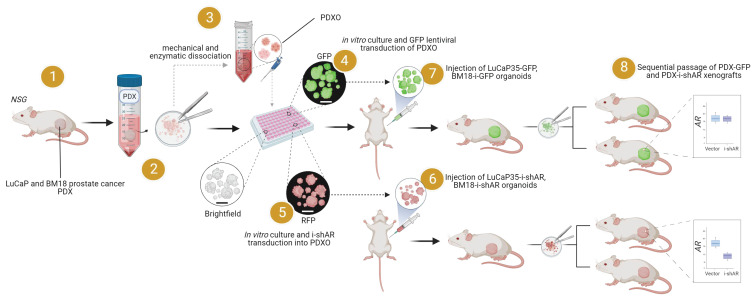
Schematic of in vivo PDX propagation, excision, ex vivo organoid generation, and subsequent genetic manipulation to force the expression of GFP and knockdown the AR in prostate cancer PDX organoids (PDXO). Created with BioRender.com.

Cells isolated from LuCaP35 and BM18 PDX models, seeded in a low attachment plate and resuspended in PCM media, all established successfully as organoid cultures (PDXOs). PDXOs were continuously maintained in vitro for up to 6 weeks with regular media exchanges. Following the successful in vitro culture of PDX lines, we tested the feasibility of genetically manipulating PDXO cultures. As proof-of-principle, PDXOs were transduced with a pGIPZ lentiviral vector containing a TurboGFP reporter and a short hairpin (sh) RNA encoding targeting firefly luciferase. LuCaP35 and BM18 lines were successfully transduced and labeled with GFP, establishing LuCaP35-GFP and BM18-GFP PDXO models (Figure 1).

### 3.2. Assessment of Tumor Formation of GFP-Labeled PDX Models In Vivo

**Figure 2:**
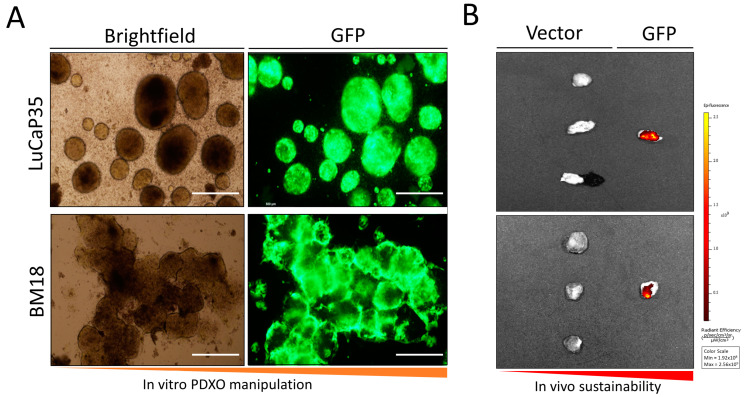
In vitro culture of organoids (PDXO) from LuCaP35 and BM18 PCa PDX and subsequent genetic manipulation. (**A**) Brightfield image of LuCaP35 and BM18 PDXO cultures following lentiviral transduction (LuCaP35-GFP and BM18-GFP). Scale bar = 500 μm. (**B**) Demonstration of stable GFP expression following in vivo passage of transduced LuCaP35-GFP PDX, and BM18-GFP PDX tumors. Representative IVIS images showing total GFP expression. Vector (non-transduced) tumors (*n* = 3) are on the left of each panel. GFP labeled tumor with persistent GFP expression (*n* = 1) are on the right of each panel.

Subcutaneous injection of BM18-GFP and LuCaP35-GFP organoid cultures resulted in robustly engraftable xenografts in NSG mice. BM18-GFP and LuCaP35-GFP-derived xenografts demonstrated stable and persistent expression of GFP when assessed by ex vivo imaging (6/6 mice; Figure 2A). Upon passaging and subsequent implantation into new host mice, stable GFP expression was demonstrated using epifluorescence microscopy (Figure 2B). Histological analyses showed that the tumor architecture and differentiation patterns of tumors grown in vehicle-treated mice were consistent with the original parental PDX line.

### 3.3. Doxycycline Inducible AR Knockdown Reduces In Vivo Tumor Growth in LuCaP35 PDX Model

**Figure 3:**
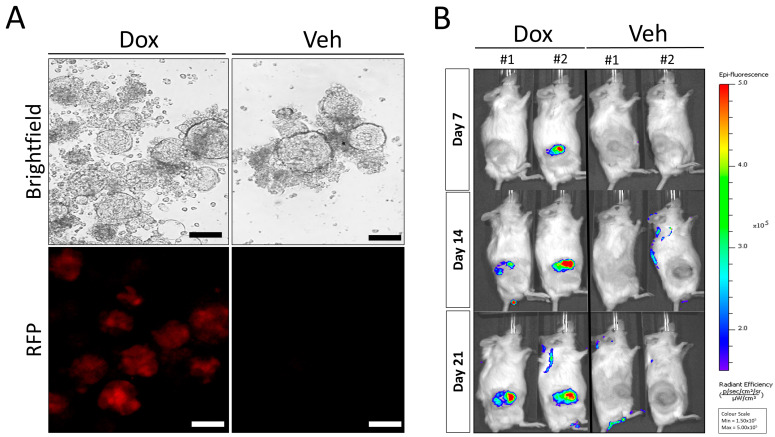
Generation of AR-depleted LuCaP35-i-shAR organoid cultures and in vivo xenograft imaging. (**A**) Representative brightfield image of LuCaP35-i-shAR organoids in PCM media in the presence of doxycycline (Dox) and corresponding fluorescence image demonstrating the absence of Turbo RFP expression (Veh). Brightfield image of doxycycline-treated LuCaP35-i-shAR organoids after 5 days and corresponding fluorescence image for Turbo RFP showing induction of expression of Turbo RFP. Scale bar = 100 μm. (**B**) In vivo imaging of LuCaP35-i-shAR tumor-bearing mice. Red fluorescent protein (RFP) fluorescent signal captured over a three-week period by IVIS Lumina Imaging System in tumor-bearing mice treated with doxycycline (Dox, *n* = 2 vs. no dox, *n* = 2) to induce expression of shAR-RFP expression.

**Figure 4:**
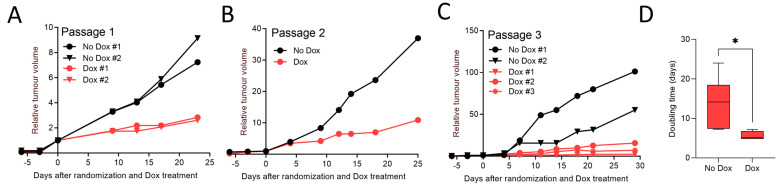
AR knockdown in LuCaP35-i-shAR xenografts reduced tumor growth and increased tumor doubling time. Tumor volume of LuCaP35-i-shAR xenografts after doxycycline (Dox) or vehicle-control treatment of mice bearing LuCaP35-i-shAR tumors at (**A**) passage 1, (**B**) passage 2, and (**C**) passage 3. (**D**) Tumor volume doubling time of all Dox-treated (*n* = 6) versus all vehicle-control-treated (*n* = 5) LuCaP35-i-shAR tumors. Mean +/− SEM, * *p* ≤ 0.05 Students *t*-test.

To demonstrate the functional utility and potential of this approach, we established a lentiviral-mediated knockdown of the AR in the androgen-sensitive LuCaP35 PDX. The AR was targeted because of the key role of this receptor in PCa growth and previously demonstrated sensitivity of this PDX model to inhibition of AR signaling [18]. We employed a doxycycline (Dox) inducible pTRIPZ lentiviral vector expressing AR-targeted shRNA (i-shAR) and vector control (i-vector) in the LuCaP35-derived organoids (Herein, denoted as LuCaP35-i-shAR and LuCaP35-i-vector). LuCaP35-i-shAR and LuCaP35-i-vector cells (and thus PDXOs) were selected for lentiviral integration by supplementing PCM media with puromycin (1 µg/mL). Turbo RFP expression was used as a surrogate marker for successful expression of shAR following induction with Dox (Figure 3A). Following the generation of LuCaP35-i-shAR, we assessed the effect of serial passaging of the PDX model on the stability of the lentivirus vector in the tumor cells. Cell viability testing revealed, on average, 55–65% viability for organoid cultures. To initiate re-transplantation into NSG mice, LuCaP35-i-shAR and LuCaP35-i-vector organoids were harvested and resuspended in 100 µL of Matrigel^TM^ prior to subcutaneous injection into NSG mice. To determine the stability of lentivirus transduction during PDX tumor serial transplantation, we first cultured the cells isolated from passage number one LuCaP35-i-shAR in vitro. We then treated the resulting organoids with Dox to assess the extent of shAR-Turbo RFP expression. Epifluorescence microscopy demonstrated persistent expression of TurboRFP. LuCaP35-i-shAR xenografts were passaged into four recipient mice, which were subsequently randomized to either Dox-treatment or vehicle-control at a 1:1 ratio once tumors reached a volume of >300 mm^3^. A significant inhibition in tumor growth in Dox-treated mice compared with the vehicle-control group was observed (Figure 3B and Figure 4A). IVIS imaging of the tumors demonstrated tumor-associated TurboRFP fluorescent signals were observed only in Dox-treated mice (Figure 3B and Figure S1), confirming the specificity of the induction of the shAR-TurboRFP expression cassette.

### 3.4. Re-Implantation of LuCaP35-i-shAR Xenografts Retain Decreases in Tumor Growth

To assess the temporal stability of lentiviral transduction and genetic manipulation of LuCaP-i-shAR cells, the PDX tissue was passaged three times independently in vivo (Figure 4A–C). Dox-exposed LuCaP35-i-shAR xenografts (*n* = 6) demonstrated a slower growth rate than the LuCaP35-i-vehicle xenografts (*n* = 5) (vehicle: 13.9 doubling time (days), Dox: 5.6 doubling time (days), *p* = 0.02) (Figure 4D). TurboRFP fluorescent signal was detected in all LuCaP35-i-shAR tumor-bearing mice treated with Dox (Supplementary Figure S1), confirming the induction of the shAR-TurboRFP expression cassette.

## 4. Discussion

We have established a novel system to culture patient-derived prostate cancer xenografts in vitro, thus enabling genetic manipulation of targets in an inducible system, reconstituted in vivo, which maintains the fidelity of the original PDX phenotype. This advance will provide increased utility for existing PDX models in prostate cancer and provide the research community with an expanded opportunity to investigate therapeutic and mechanistic targets in a more complex in vivo model system.

Advances in genomic and transcriptomic technologies have revolutionized our knowledge of molecular profiling of PCa and the pathways involved in disease formation and progression. However, investigating the functionality and impact of a particular target by genetic manipulation has been hindered by a lack of representative preclinical PCa models. After several decades of intensive but unproductive efforts to establish clinically representative PCa cell lines, PDXs have become widely accepted as alternative translational models available for preclinical oncology research. Indeed, several PCa PDX models (such as LuCaP35 and BM18 used here) have been established by different laboratories [18,19,20,21,22]. However, due to several limitations such as the lack of a reliable method for in vitro cell culture of PDXs and difficulties with genetic manipulation of PDX-derived cells, they have been restricted to in vivo use. These limitations make PDXs costly and not readily applicable for high-throughput drug screening or targeting of specific genes of interest. Like patient-derived prostate cells [24], we have demonstrated that while prostate PDXs are difficult to culture in monolayer, they can be grown in vitro as organoids using a modified culture media. 

Our results indicate we can successfully label organoids of prostate origin with a fluorescent protein marker, enabling in vitro and in vivo fluorescent imaging. The labeled organoids re-established xenografts after subcutaneous implantation and stably retained the fluorescent label. Stable fluorescent labeling of xenograft cells enables non-invasive monitoring of tumors and thereby enables longitudinal and endpoint measurement of tumor growth, treatment response to therapy, and the formation of metastasis in vivo. The introduction of such traceable markers (e.g., GFP, RFP) and either overexpression or knockdown of specific genes enables researchers to use PDXs to investigate fundamental questions about tumor formation, growth, and metastasis in a similar way to the limited number of established cell lines that have been used [31]. Notably, our system is distinct as it is the first to describe its application in prostate cancer PDXs. Unlike prior studies utilizing CRISPR-Cas9 technology to manipulate solid tumor PDXs, our method employs inducible short hairpin RNA (shRNA), which is widely used in experimental settings for gene knockdown. This approach offers an accessible, rapid, and cost-effective gene knockdown with potentially fewer off-target effects and a simpler experimental setup, setting it apart from other genetically programmable models [32].

We were also able to genetically manipulate the organoids by lentiviral transduction with an inducible system. The resulting LuCaP35-i-shAR cells formed tumors when implanted subcutaneously in NSG mice and retained the lentiviral vector expressing TurboRFP gene and AR shRNA. We were also able to successfully confirm the utility of an inducible system in an in vivo model to target a gene of interest. Using this system, we were able to image the mice and confirm the activation of lentiviral vector and an associated biological response. 

## 5. Conclusions

Induction of knockdown of AR in LuCaP35 by Dox treatment resulted in a reduction in tumor growth and demonstrated the dependency of LuCaP35 cells on the AR signaling pathway. The methodology we have described in this study to generate the LuCaP35-i-shAR model provides researchers with the capacity to generate preclinical PDX screening models for solid tumors to recapitulate the genetic alterations identified from genomic and transcriptomic profiling in patient samples. This may lead to being able to test and distinguish between driver and passenger mutations. Furthermore, this method may be useful to identify patients who could respond to targeted, novel, or repurposed therapies, a major goal of translational research and precision medicine. Through organoid culture, it is possible to rapidly test and investigate multiple drugs in vitro prior to initiating in vivo studies, saving time, and reducing costs of therapeutic screening significantly, as well as ultimately improving patient outcomes.

## Data Availability

The data presented in this study are available on request from the corresponding author.

## Supplementary Materials

The following supporting information can be downloaded at: https://www.mdpi.com/article/10.3390/curroncol30110683/s1, Table S1: Composition of prostate cancer medium (PCM); Figure S1: In vivo imaging of serially passaged LuCaP35-i-shAR tumor bearing mice (passage number 2 and 3).
