# Supplementary material for: Introduction of Androgen Receptor Targeting shRNA Inhibits Tumor Growth in Patient-Derived Prostate Cancer Xenografts
Source: Curr Oncol. 2023 Oct 24;30(11):9437–47. doi: 10.3390/curroncol30110683 (PMC10670201; doi:10.3390/curroncol30110683)
Supplement: Supplementary file 1 [file curroncol-30-00683-s001.zip › curroncol-2664554-supplementary.pdf]

Current Oncology

MDPI

Article

Introduction of Androgen Receptor Targeting shRNA Inhibits Tumor Growth in Patient-Derived Prostate Cancer Xenografts

Patrick B. Thomas [1,2,3], Saeid Alinezhad [1,2], Andre Joshi [1,2,3,4], Katrina Sweeney [1,2], Brian W. C. Tse [5], Gregor Tevz [1,2], Stephen McPherson [1,2], Colleen C. Nelson [1,2,6], Elizabeth D. Williams [1,2,3,6,*,†] and Ian Vela [1,2,3,4,†]

1 School of Biomedical Sciences at Translational Research Institute (TRI), Faculty of Health, Queensland University of Technology (QUT), Brisbane, QLD 4102, Australia; pb.thomas@qut.edu.au (P.B.T.); saeid.alinezhad@qut.edu.au (S.A.); andre.joshi@health.qld.gov.au (A.J.); katrina.sweeney@qut.edu.au (K.S.); gregor.tevz@qut.edu.au (G.T.); stephen.mcpherson@uq.edu.au (S.M.); colleen.nelson@qut.edu.au (C.C.N.); ian.vela@health.qld.gov.au (I.V.)
2 Australian Prostate Cancer Research Centre—Queensland, Brisbane, QLD 4102, Australia
3 Queensland Bladder Cancer Initiative (QBCI), Brisbane, QLD 4102, Australia
4 Department of Urology, Princess Alexandra Hospital, Brisbane, QLD 4102, Australia
5 Preclinical Imaging Facility, Translational Research Institute (TRI), Brisbane, QLD 4102, Australia; brian.tse@tri.edu.au
6 Centre for Genomics and Personalised Health, Queensland University of Technology (QUT), Brisbane, QLD 4000, Australia
* Correspondence: ed.williams@qut.edu.au
† These authors contributed equally to this work.

Table S1. Composition of prostate cancer medium (PCM).

Additive	Final Conc.	Stock Conc.	Solvent	Company	Catalogue no.
R-spondin conditioned media	10% v/v	Conditioned media	Advanced DMEM/F-12	/	/
Noggin conditioned media	10% v/v	Conditioned media	Advanced DMEM/F-12	/	/
Glutamax	2 mM	1 M	NaCL	Invitrogen	35050-061
EGF	5 ng/ml	0.5 mg/ml	PBS/0.1% BSA	Sigma	SRP3196-500UG
FGF-10	10 ng/ml	100 µg/ml	PBS/0.1% BSA	Peprotech	100-26-1000
FGF-2	5 ng/ml	50 µg/ml	PBS/0.1% BSA	Peprotech	100-18B
HEPES	10 mM	1000x diluted	NaCl/NaHPO4 buffered solution	Gibco	/
Nicotinamide	10 mM	1 M	ddH_20	Sigma	NO636
N-acetyl-L-cysteine	1.25 mM	500 mM	ddH_20	Sigma	A9165-25G
A83-01	0.5 µM	500 µM	DMSO	BioScientific	2939/10
SB202190	10 µM	50 mM	DMSO	Selleckchem	S1077-SEL-100MG
Y27632	10 µM	100 mM	ddH_20	Selleckchem	S1049-SEL-50MG
B27 additive	1X	50x diluted	/	Gibco	17504-044
Primocin	1:100 v/v	/	/	InvivoGen	ANT-PM-1
DHT	1 nM	1 µM	Ethanol	/	/

DHT: dihydrotestosterone; EGF: epidermal growth factor; FGF: fibroblast growth factor; HEPES: 4-(2-hydroxyethyl)-1-piperazineethanesulfonic acid.

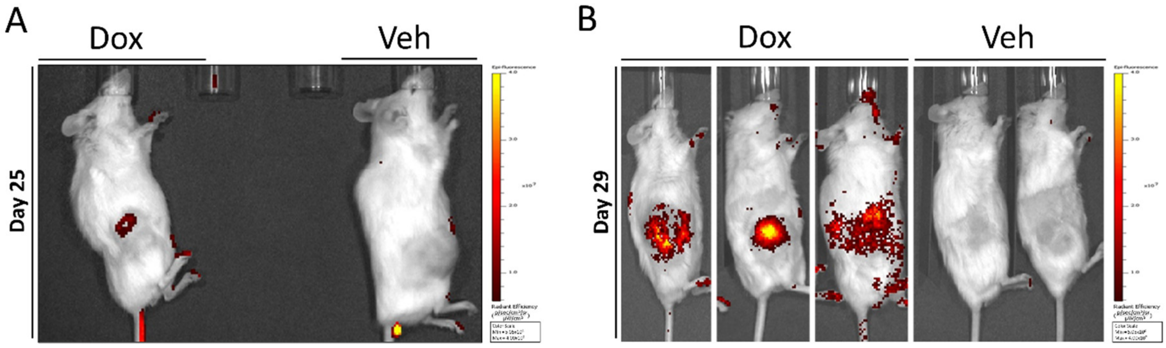

Figure S1. ***In vivo*** **imaging of serially passaged LuCaP35-i-shAR tumor bearing mice (passage number 2 and 3).** **(A)** IVIS *in vivo* imaging of LuCAP35-i-shAR xenograft passage 2 after 25 days of Dox treatment and **(B)** LuCAP35-i-shAR xenograft passage 3 after 29 days of Dox treatment. No fluorescence signal was detected in the tumors of vehicle-control-treated mice.
